# Recover your smile: Effects of a beauty care intervention on depressive symptoms, quality of life, and self‐esteem in patients with early breast cancer

**DOI:** 10.1002/pon.4957

**Published:** 2018-12-21

**Authors:** Anna Richard, Nadia Harbeck, Rachel Wuerstlein, Frank H. Wilhelm

**Affiliations:** ^1^ Division of Clinical Psychology, Psychotherapy, and Health Psychology, Department of Psychology University of Salzburg Salzburg Austria; ^2^ Center for Cognitive Neuroscience University of Salzburg Salzburg Austria; ^3^ Department of Gynecology and Obstetrics Breast Center and CCC Munich Munich Germany

**Keywords:** appearance‐related side effects, breast cancer, beauty care intervention, cancer distress, oncology, patient‐reported outcomes, psycho‐oncology, psychosocial intervention, supportive care

## Abstract

**Objective:**

Medical cancer treatment is often accompanied by appearance‐related side effects such as hair loss, skin irritation, and paleness, which can subsequently lead to psychosocial distress. Initial evidence suggests that beauty care interventions may reduce distress and improve quality of life (QoL), body image, and self‐esteem immediately.

**Methods:**

We investigated the effects of a brief beauty care intervention on self‐reported symptoms of depression, quality of life, body image, and self‐esteem in 39 female primary breast cancer patients with appearance‐related treatment side effects. Patients were randomly assigned either to an immediate intervention group (IG) or to a wait‐list control (WL). The intervention consisted of a single‐session group makeup workshop, a photo shooting, and of receiving professionally edited portrait and upper‐body photos.

**Results:**

While groups did not differ regarding any measure at the pretreatment baseline assessment, IG patients reported less symptoms of depression, higher QoL, and higher self‐esteem compared with baseline and compared with WL. Follow‐up at 8 weeks indicated moderate stability of these improvements.

**Conclusions:**

In contrast to previous research, results indicate beneficial short‐term and midterm effects of beauty care on psychological outcomes in patients with early breast cancer. These results emphasize the utility of this type of brief, low‐cost intervention in women undergoing medical cancer treatment in order to improve their well‐being.

## 1 BACKGROUND

The majority of breast cancer patients are faced with appearance‐related side effects (eg, loss of scalp hair and eyebrows or eyelashes, irritated skin, or scar formation) while undergoing medical cancer treatment. These treatment‐induced changes in appearance have been considered a primary reason for distress^1^, ^2^ and have been linked to decreases in a range of psychological outcomes, including body image,^3^ psychosocial well‐being, and quality of life.^4^, ^5^ Importantly, higher distress and a decrease in quality of life were found to negatively affect cancer progression and survival rates,^6^, ^7^ pointing towards the need for adequate psychosocial interventions targeting appearance‐related side effects to improve psychological outcomes.

Over the past decades, numerous group‐based psychosocial education programs have been put forward to overcome psychological distress related to breast cancer treatment.^8^ One of these interventions is skin and camouflage treatment, which directly addresses changes in appearance by teaching skin care and applying makeup. The effectiveness of makeup to improve psychological outcomes has been supported recently in patients with head and neck cancer,^9^ showing that symptoms of depression and anxiety were reduced, and body image was increased up to 3 months after treatment. Similarly, beauty care was found to immediately decrease anxiety and increase self‐image in a heterogeneous sample of cancer patients, particularly in those reporting appearance‐related side effects at the time of intervention.^10^

Research on the immediate and short‐term effects of beauty care interventions in breast cancer patients, however, is less clear: It was consistently found that perception of attractiveness was improved, and symptoms of depression and anxiety were immediately decreased by beauty care.^11^, ^12^, ^13^ While these results support the idea that beauty care has beneficial effects on psychological outcomes on the short term, no conclusions can yet be drawn about their specificity, reliability, and stability because of a lack of control groups (including randomized assignment), low‐sample sizes, and short or absent follow‐ups. The few studies that examined both short‐ and longer‐term effects of beauty care produced mixed findings. For example, although beauty care treatment increased body image satisfaction after surgery, these improvements did not persist to 3 months later.^14^ Similarly, immediate decreases in distress and increases in stress coping have recently been documented,^15^ but no differences were found at 1‐month follow‐up. Initial evidence indicates, however, that beauty care may improve facets of quality of life midterm to the effect of a higher self‐confidence and optimism even after the end of radiotherapy.^16^

Previous research suggests that beauty care for reducing appearance‐related side effects has an immediate beneficial effect on a range of psychological outcomes (eg, symptoms of depression, quality of life, self‐esteem, and body image satisfaction). However, results are still inconclusive regarding the stability of these improvements. In the current study, 39 primary breast cancer patients with appearance‐related side effects were randomized to an immediate intervention group (IG) or wait‐list control group (WL) and completed trait questionnaires on symptoms of depression, quality of life, body image, and self‐esteem at four measurement points (ie, baseline and 2, 4, and 8 weeks after baseline). The intervention consisted of a single‐session group makeup workshop. In addition to the methodological improvements compared with previous studies, we also incorporated a portrait and upper‐body photo shooting. Photos were also professionally edited and sent to the patients, which may have a favorable effect on study outcomes as well. On the basis of previous findings, we hypothesized that there would be immediate and short‐term decreases in symptoms of depression and increases in quality of life, body image, and self‐esteem for IG but not for WL. Furthermore, we expected stability of improvements on psychological outcomes after 8 weeks.

## 2 METHODS

### 2.1 Participants

**Figure 1:**
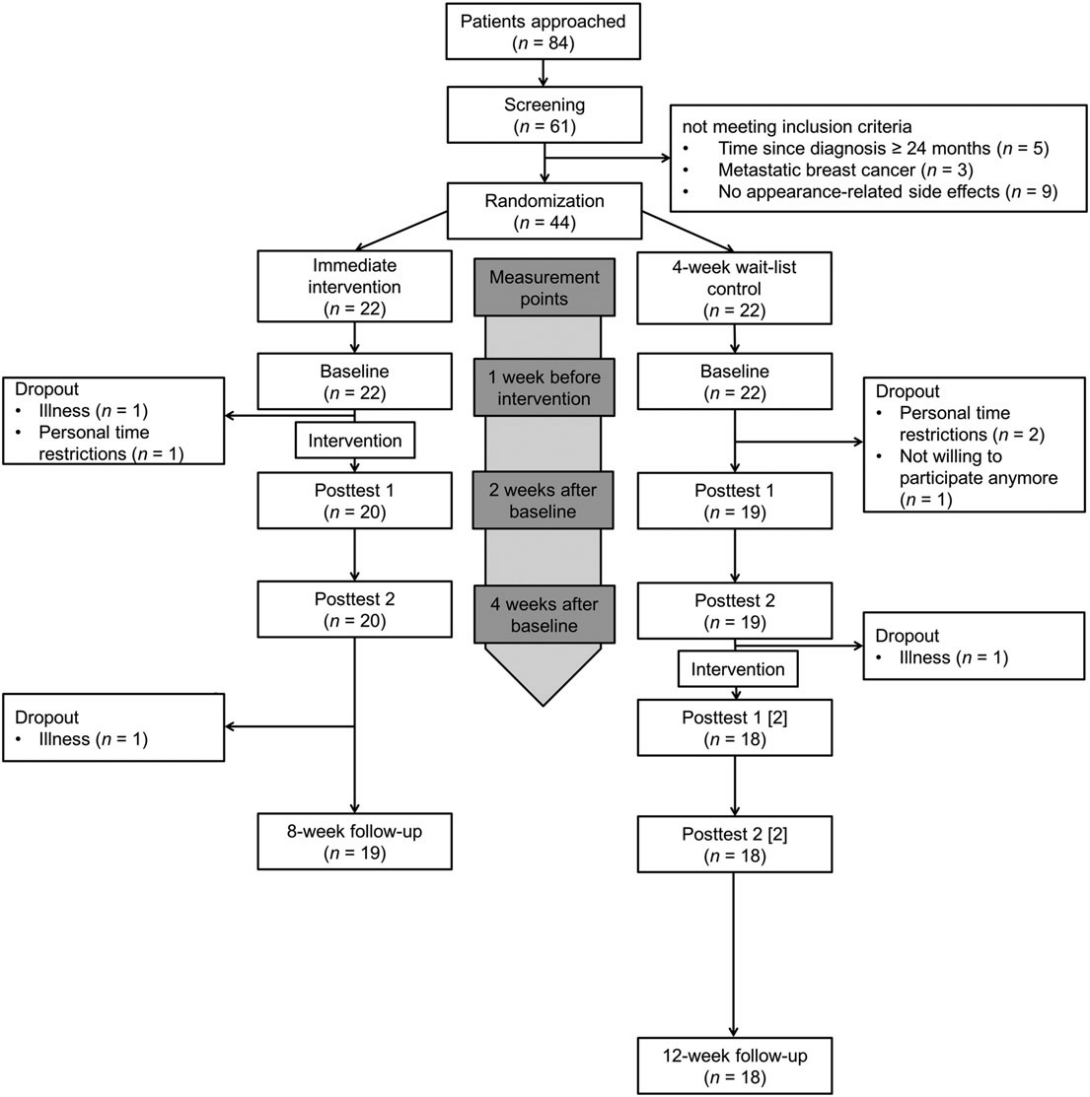
Flow of participants during the study. Wait‐list control entered active treatment 5 weeks after baseline and between‐group comparisons were conducted for baseline, posttest 1, and posttest 2. Follow‐up assessment was used to examine the stability of treatment effects 8 weeks after baseline. Numbers in squared brackets indicate that the WL completed posttest 1 and 2 for a second time after receiving the intervention

Eighty‐four women with a diagnosis of early breast cancer were recruited through in‐hospital advertisement at the Breast Center of the Ludwig Maximilian University of Munich, Germany. Sixty‐one patients were screened for the following inclusion criteria: primary breast cancer, 18 years old and above, reporting appearance‐related side effects of cancer treatment (eg, irritated or pale skin, loss of scalp hair, eyebrows, or eyelashes), and time since diagnosis less than 24 months. Forty‐four eligible participants were randomly allocated to either IG or WL (see Figure 1 for participant flow and dropout). Descriptive statistics of the final sample are shown in Table 1. The study was approved by the ethics committees of the University of Salzburg (approval number: 24/2014) and the Medical Faculty of the University of Munich (approval number: 563‐14), and all participants provided written informed consent before study entry.

**Table 1:** Demographic and clinical characteristics of immediate intervention group and wait‐list control group at pre‐treatment baseline

	Immediate Intervention Group (*n* = 20)	Wait‐list Control Group (*n* = 19)	*P* values
Age (years)†	39.6 (9.35)	37.4 (9.60)	0.468
Time since diagnosis (months)†	6.82 (5.37)	7.72 (6.01)	0.452
Education (years)†	15.3 (2.83)	14.0 (3.32)	0.212
Marital status‡			0.709
Single	4 (20.0%)	3 (15.8%)	
Partnership	4 (20.0%)	7 (36.8%)	
Married	9 (45.0%)	7 (36.8%)	
Divorced/separated/widowed	2 (10.0%)	2 (10.5%)	
Children (yes)‡	12 (60.0%)	10 (52.6%)	0.643
Job status‡			0.935
Sick leave	16 (80.0%)	15 (78.9%)	
Working	4 (20.0%)	4 (21.1%)	
Monthly income‡			0.845
<1000€	6 (30.0%)	7 (36.8%)	
1000‐2000€	8 (40.0%)	5 (31.6%)	
>2000€	6 (25.0%)	6 (26.3%)	
Tumor size‡			0.431
<2 cm	6 (30.0%)	8 (42.1%)	
2‐5 cm	14 (60.0%)	11 (57.9%)	
Radiation therapy (yes)‡	4 (20.0%)	6 (31.6%)	0.480
Chemotherapy (yes)‡	18 (90.0%)	16 (84.2%)	0.661
Mastectomy (yes)‡	5 (25.0%)	8 (42.1%)	0.257

† Means (and standard deviations) are displayed.

‡ Absolute counts (and percentages) are displayed.

### 2.2 Questionnaire measures

*State‐Trait Depression Scales (STDS)*. The STDS^17^ was used to measure current symptoms of depression. It asks about the presence of depressive symptoms (eg, “I feel depressed.”; dysthymia subscale; five items) and the absence of positive affect (eg, “I feel safe.”; euthymia subscale; five items). Items were rated on a 4‐point Likert‐type scale (0 [*not at all*] to 3 [*very much*]). Total scores can range from 0 to 30, and scores on the dysthymia and euthymia subscales can range from 0 to 15, respectively. Lower scores indicate less current symptoms of depression. Internal consistencies in the current sample ranged between *α* = 0.753 and *α* = 0.848 across measurement points.

*Centre for Epidemiological Studies Depression Scale (CESD)*. The CESD^18^ was used to measure symptoms of depression in general according to the past week. The scale consists of 20 items (eg, “I was bothered by things that usually don't bother me.”) rated on a 4‐point Likert‐type scale (0 [*rarely/none of the time (less than one day)*] to 3 [*most/all of the time (five to seven days)*]). Total scores can range from 0 to 60, and lower scores indicate less symptoms of depression in general. Internal consistencies in the current sample ranged between *α* = 0.897 and *α* = 0.930 across measurement points.

*Functional Assessment of Cancer Therapy‐Breast (FACT‐B)*. The FACT‐B^19^ was used to measure quality of life for patients with breast cancer. The subscales physical (eg, “I'm bothered by side effects of treatment.”; seven items), social/familial (eg, “I feel close to my friends.”; seven items), emotional (eg, “I feel sad.”; six items), and functional well‐being (eg, “I'm enjoying the things I usually do for fun.”; seven items), and the breast cancer subscale (eg, “I feel sexually attractive.”; 10 items) were rated on a 5‐point Likert‐type scale (0 [*not at all*] to 4 [*very much*]). Total scores can range from 0 to 148, and higher scores indicate higher quality of life in general and on the subscales, respectively. Internal consistencies in the current sample ranged between *α* = 0.926 and *α* = 0.944 across measurement points.

*Body Image Scale (BIS)*. The BIS^20^ was used to measure the impact of appearance‐related side effects of cancer treatment on body image. The scale consists of 10 items (eg, “Have you felt less physically attractive as a result of your disease or treatment?”) rated on a 4‐point Likert‐type scale (0 [*not at all*] to 3 [*very much*]). Total scores can range from 0 to 30, and higher scores indicate higher body image satisfaction. Internal consistencies in the current sample ranged between *α* = 0.852 and *α* = 0.914 across measurement points.

*Rosenberg Self‐Esteem Scale (RSES)*. The revised version of the RSES^21^ was used to measure self‐esteem. The scale consists of 10 items (eg, “I take a positive attitude toward myself.”) rated on a 4‐point Likert‐type scale (0 [*strongly disagree*] to 3 [*strongly agree*]). Total scores can range from 0 to 30, and higher scores indicate higher self‐esteem. Internal consistencies in the current sample ranged between *α* = 0.786 and *α* = 0.836 across measurement points.

*Intervention credibility*. Participants were asked whether they deemed the approach of the intervention to improve well‐being in breast cancer patients as reasonable, whether they felt confident that the intervention will improve their own well‐being, whether they would recommend the intervention to a friend suffering from cancer, and as control item, whether they felt confident that the intervention will help to overcome general health problems (eg, insomnia and headaches). Items were rated on an 11‐point Likert‐type scale (0 [*not at all*] to 10 [*very much*]).

### 2.3 Procedure

Breast cancer patients with appearance‐related side effects of cancer treatment were informed about the study by the breast care nurses. Interested patients were screened for eligibility and were then randomly allocated to IG and WL, respectively. All patients completed a set of questionnaires at four times: baseline, posttest 1 (2 weeks after baseline), posttest 2 (4 weeks after baseline), and follow‐up (8 weeks after baseline). Five weeks after baseline, WL entered active treatment and passed through the same procedure as IG, that is, receiving the intervention (5 weeks after baseline) and completing posttest 1 (6 weeks after baseline), posttest 2 (8 weeks after baseline), and follow‐up (12 weeks after baseline). Demographic and clinical variables were collected at baseline.

The intervention for IG took place 1 week after baseline and consisted of a single 4‐hour makeup workshop and photo shoot at a makeup school. Participants completed the STDS immediately before the intervention. They were then trained by professional beauty specialists and were introduced to useful skills, including skin care and the use of makeup to cover appearance‐related side effects. Two different makeups were applied, after which participants were encouraged to participate in a portrait and upper‐body photo shoot, respectively. Participants then completed the STDS for a second time and were asked to rate the credibility of this intervention. Finally, they received their professionally edited photos 2 weeks after intervention by e‐mail (after completing posttest 1 but before completing posttest 2). All makeup workshops were conducted as a part of the publicly available “Recover Your Smile” program (www.recoveryoursmile.org).

### 2.4 Statistical analyses

**Figure 2:**
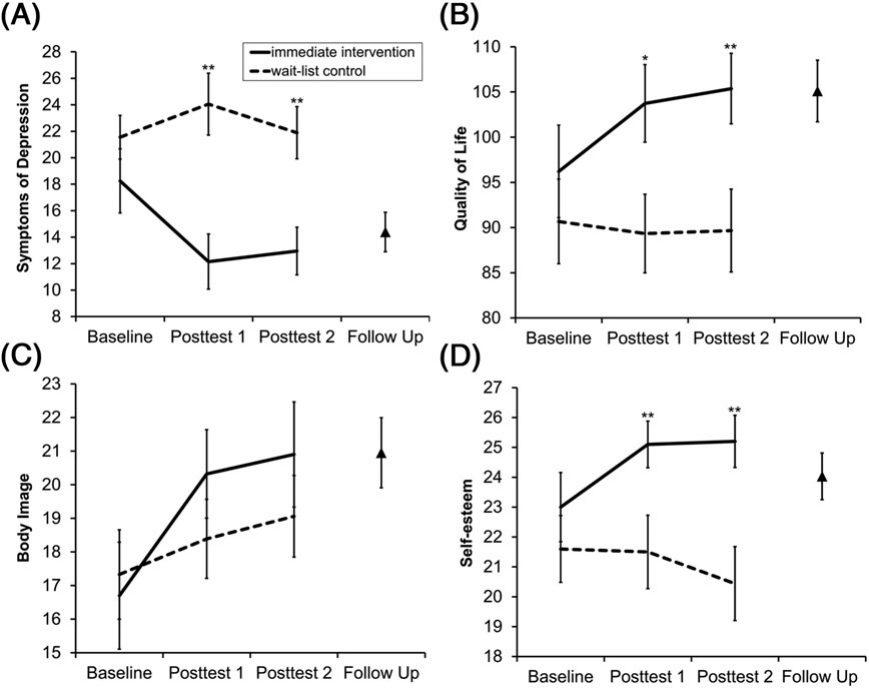
Short‐term and midterm treatment effects as a function of *Group* (immediate intervention [IG], wait‐list control group [WL]), and *Time* (baseline [pretreatment], posttest 1 [after IG, but not WL, had completed the makeup workshop], and posttest 2 [after IG, but not WL, had received the photos]) for A, symptoms of depression, B, breast cancer‐related quality of life, C, body image, and D, self‐esteem. Groups did not differ in any measure before treatment, whereas IG (solid lines) compared with WL (dashed lines) showed treatment‐related improvements of depressive symptoms, quality of life, and self‐esteem. Body satisfaction increased in both groups independent of treatment. Triangles indicate midterm treatment effects for follow‐up 8 weeks after baseline. Error bars represent standard errors of the mean. Asterisks indicate significant Bonferroni‐corrected between‐group differences at ** *P* < 0.010 or * *P* < 0.050

Pearson's χ^2^ tests or Fisher's exact tests for categorical variables and independent samples *t* tests for continuous variables were conducted to test for between‐group differences in demographic and clinical variables at baseline (Table 1). Paired samples *t* tests were used to test for changes in current symptoms of depression immediately before and after the intervention. Analyses of variance for repeated measures with *Group* (IG and WL) as between‐subject factor and *Time* (baseline, posttest 1, posttest 2) as within‐subject factor were calculated to examine short‐term treatment effects on symptoms of depression, quality of life, body image, and self‐esteem. Significant *Group* × *Time* interactions were followed up with Bonferroni‐corrected pairwise comparisons using mean differences (*MD*) between IG and WL at baseline, posttest 1 and posttest 2, respectively. Figure 2 displays treatment‐related improvements of depressive symptoms (A), quality of life (B), body image (C), and self‐esteem (D) for IG and WL at baseline, posttest 1, posttest 2, and follow‐up. Primary analyses were conducted with the per‐protocol sample, comprising patients who completed baseline, posttest 1, and posttest 2 (IG: *n* = 20, WL: *n* = 19). Further analyses were conducted with the intention‐to‐treat sample, comprising all patients who underwent randomization irrespective of their dropout (IG: *N* = 22, WL: *N* = 22).* Paired samples *t* tests were used to examine midterm treatment effects between posttest 2 and follow‐up for IG and—as WL also received the treatment and completed posttest 1 and posttest 2 a second time—for the whole sample, respectively. Effect sizes are reported as partial eta squared (η_p_^2^) or (baseline‐corrected) Cohen's *d*.

## 3 RESULTS

### 3.1 Immediate treatment effects

*Intervention credibility*. Participants deemed the approach of the intervention to improve well‐being in breast cancer patients as highly reasonable (*M* = 9.29, *SD* = 1.01). They also felt confident that the intervention would rather improve their own well‐being (*M* = 8.87, *SD* = 1.12) than help to overcome general health problems (*M* = 5.68, *SD* = 2.52). Patients also stated that they would recommend the intervention to a friend suffering from cancer (*M* = 8.87, *SD* = 1.46).

*Current symptoms of depression*. Current depressive symptoms decreased from the beginning (*M* = 20.0, *SD* = 5.03) to the end (*M* = 15.1, *SD* = 3.33) of the intervention, *t*_(37)_ = 7.90, *P* < 0.001, *d* = 1.41. These changes applied to both the dysthymia subscale (pre: *M* = 8.16, *SD* = 2.58; post: *M* = 5.95, *SD* = 1.39) and the (negatively coded) euthymia subscale (pre: *M* = 11.8, *SD* = 3.14; post: *M* = 9.16, *SD* = 2.58), both *t*s_(37)_ ≥ 5.26, *P*s < 0.001 and *d*s ≥ 0.92.

### 3.2 Short‐term and midterm treatment effects

*Symptoms of depression*. There was no main effect of *Time*, *F*_(2,74)_ = 1.64, *P* = 0.201, η_p_^2^ = 0.042, but a main effect of *Group*, *F*_(1,37)_ = 9.14, *P* = 0.005, η_p_^2^ = 0.198. This main effect, however, was modulated by a *Group* × *Time* interaction, *F*_(2,74)_ = 3.45, *P* = 0.037, η_p_^2^ = 0.085: No differences between groups were found at baseline, *MD* = −4.12, *P* = 0.229, (95% CI, −10.9‐2.70), whereas depressive symptoms were lower for IG than for WL both at posttest 1, *MD* = −11.9, *P* = 0.001, (95% CI, −18.6 to −5.19), and at posttest 2, *MD* = −8.10, *P* = 0.006, (95% CI. −13.7 to −2.47). Baseline‐corrected effect sizes indicated that these between‐group differences were of medium‐to‐large magnitude (*d* = −0.83 at posttest 1; *d* = −0.45 at posttest 2). Depressive symptoms did not differ between posttest 2 and follow‐up (IG: *t*_(18)_ = 0.28, *P* = 0.785, *d* = 0.07; whole sample: *t*_(36)_ = 0.45, *P* = 0.657, *d* = 0.07), indicating that improvements of depressive symptoms remained stable after 4 weeks (Figure 2A).

*Quality of life*. There was no main effect of *Time*, *F*_(2,74)_ = 2.23, *P* = 0.114, η_p_^2^ = 0.057, but a main effect of *Group*, *F*_(1,37)_ = 5.17, *P* = 0.029, η_p_^2^ = 0.123. This main effect, however, was modulated by a *Group* × *Time* interaction, *F*_(2,74)_ = 3.85, *P* = 0.026, η_p_^2^ = 0.094: No differences between groups were found at baseline, *MD* = 7.85, *P* = 0.271, (95% CI, −6.38‐22.1), whereas general quality of life scores were higher for IG than for WL both at posttest 1, *MD* = 16.8, *P* = 0.010, (95% CI, 4.34‐29.2), and at posttest 2, *MD* = 16.7, *P* = 0.009, (95% CI, 4.42‐29.1). Baseline‐corrected effect sizes indicated that these between‐group differences were of medium‐to‐large magnitude (*d* = 0.81 at posttest 1; *d* = 0.67 at posttest 2). Quality of life did not differ between posttest 2 and follow‐up (IG: *t*_(18)_= −1.66, *P* = 0.114, *d* = −0.42; whole sample: *t*_(36)_ = −1.21, *P* = 0.235, *d* = −0.20), indicating that improvements of quality of life remained stable after 4 weeks (Figure 2B). However, only the breast cancer subscale revealed a *Group* × *Time* interaction, *F*_(2,74)_ = 7.83, *P* = 0.001, η_p_^2^ = 0.157, whereas interactions for the other subscales were not significant (all *F*s ≤ 2.53, *P*s ≥ 0.086, and η_ps_^2^ ≤ 0.064).

*Body image*. There was no main effect of *Group*, *F*_(1,37)_ = 1.56, *P* = 0.220, η_p_^2^ = 0.040, but a main effect of *Time*, *F*_(2,74)_ = 7.41, *P* = 0.001, η_p_^2^ = 0.167, indicating that both groups experienced increases in body image satisfaction from baseline to posttest 1, *MD* = −2.18, *P* = 0.011, (95% CI, −3.93 to −0.42), and from baseline to posttest 2, *MD* = −2.57, *P* = 0.007, (95% CI, −4.55 to −0.59). The *Group* × *Time* interaction was not significant, *F*_(2,74)_ = 1.38, *P* = 0.257, η_p_^2^ = 0.036, indicating that improvements of body image were independent of the treatment (Figure 2C).

*Self‐esteem*. There was no main effect of *Time*, *F*_(2,74)_ = 1.90, *P* = 0.157, η_p_^2^ = 0.049, but a main effect of *Group*, *F*_(1,37)_ = 7.24, *P* = 0.011, η_p_^2^ = 0.164. This main effect, however, was modulated by a *Group* × *Time* interaction, *F*_(2,74)_ = 3.54, *P* = 0.034, η_p_^2^ = 0.087: No differences between groups were found at baseline, *MD* = 1.57, *P* = 0.341, (95% CI, −1.72‐4.86), whereas self‐esteem was higher for IG than for WL both at posttest 1, *MD* = 4.17, *P* = 0.005, (95% CI, 1.31‐7.04), and at posttest 2, *MD* = 4.83, *P* = 0.002, (95% CI, 1.84‐7.82). Baseline‐corrected effect sizes indicated that these between‐group differences were of medium magnitude (*d* = 0.68 at posttest 1; *d* = 0.72 at posttest 2). Self‐esteem did not differ between posttest 2 and follow‐up (IG: *t*_(18)_= − 1.26, *P* = 0.225, *d* = −0.29; whole sample, *t*_(36)_ = −0.30, *P* = 0.765, *d* = −0.05), indicating that improvements on self‐esteem remained stable after 4 weeks (Figure 2D).

## 4 CONCLUSIONS

This study aimed at investigating the immediate, short‐term, and midterm effects of a beauty care intervention on symptoms of depression, quality of life, self‐esteem, and body image in patients with early breast cancer. Two and 4 weeks after baseline, patients of IG reported fewer symptoms of depression, higher quality of life, and higher self‐esteem as compared with baseline and compared with WL, respectively. Both groups reported increases in body image, irrespective of intervention. Follow‐up at 8 weeks indicated moderate stability of the improvements.

Immediate improvements in psychological outcomes have been reported consistently in studies investigating the effects of beauty care interventions in breast cancer patients. However, it has been less clear whether these interventions only have a short‐term or also a longer‐term psychological effect, as prior results were often limited to treatment effects, which were investigated immediately after the intervention (eg, on the same day) or to studies that did not implement a randomized control group design. In contrast to previous studies (eg,^15^, ^16^), the current study was the first to consistently demonstrate midterm improvements on a range of psychological outcomes. As previous studies varied to a large degree regarding the examined follow‐up intervals, this may be due to methodological differences: Some studies did not include follow‐up assessments,^10^, ^11^ thus leaving it unclear if the intervention had any lasting effect at all; others used quite long follow‐up intervals (eg, several months^16^) with no in‐between measurement points and indicated no lasting treatment effect. Because individual outcome during the course of medical breast cancer treatment and environmental variables like social support can strongly vary across individuals and time, these variations are likely to mask the effects of psychosocial interventions in long‐term assessments. In addition to these methodological improvements, our study used a novel supplementary photo shooting that may have increased efficacy of the beauty care intervention as well. As a result, participants could visualize the bodily appearance effects of the beauty care intervention for a second time and share these professionally edited photos with friends and family via social media and other channels, thereby likely fostering increases in self‐esteem. Further research is needed to examine the additive effects of photography to improve psychological outcomes compared with beauty care alone.

Results of the current study confirm that participation in a beauty care intervention addressing appearance‐related treatment side effects went along with improvements on a variety of psychological outcomes, except for body image satisfaction. Contrary to our expectation, the current intervention had no differential effect on body image as both groups reported similar increases over time. While these results indeed were in line with one recent study,^15^ positive effects on body image have been documented previously in breast cancer patients receiving a beauty care intervention within 1‐week postsurgery,^14^ pointing towards the need for interventions to be carried out close to the onset of cancer treatment to have an effect on body image satisfaction as well. Thus, body image effects of the current intervention may be attenuated by the longer duration between diagnosis and intervention.

### 4.1 Study limitations

Interpretation of results is based on a sample of primary breast cancer patients reporting appearance‐related side effects, which limits generalizability to patients with metastases or other cancer sites or to patients not suffering from appearance changes. Furthermore, the current study consisted of a single‐session makeup workshop. Future studies may investigate whether multisession makeup workshops may provide additional psychosocial benefits. Finally, results of the current study exclusively relied on quantitative data (ie, standardized self‐report measures). Thus, future studies may also compile qualitative data (eg, interviews) in order to gain insights into the participants' subjective experiences as well as the underlying mechanisms of improvements.

### 4.2 Clinical implications and conclusions

Results of the current study show that psychological outcomes can be improved through a relatively brief and low‐cost beauty care intervention in breast cancer patients reporting appearance‐related treatment side effects. Specifically, the current intervention decreased symptoms of depression and improved quality of life and self‐esteem immediately and in the midterm. As psychological variables have previously been linked to long‐term medical outcomes (eg, mortality rates),^22^, ^23^ beauty care as an integral part of supportive breast cancer treatment may promote medical and psychological adjustment to disease. Therefore, future studies may investigate whether beauty care interventions could have beneficial effects on medical outcomes and whether these are mediated by improvements in psychological (eg, depression and self‐esteem), behavioral (eg, treatment compliance), psychophysiological (eg, [para‐]sympathetic activation),^24^ or psychoendocrinological (eg, cortisol and oxytocin) variables.

As appearance‐related side effects are commonly experienced by the majority of breast cancer patients, the current intervention is recommended to these patients to cope with the distress during medical treatment. In contrast to other supportive care treatments, the current intervention takes place outside a hospital setting and may, thereby, foster distraction from inpatient cancer treatment. To conclude, by demonstrating positive effects on a range of psychological outcomes and by yielding a high credibility among patients, the current study suggests the use of this type of brief, low‐cost psychosocial intervention in women undergoing medical breast cancer treatment.

## CONFLICT OF INTEREST

The authors declare no conflict of interest.

## ENDNOTE

*
Including all randomized patients in an intention‐to‐treat analyses did not change pattern or significance of the *Group* × *Time* interactions (symptoms of depression: *P* = 0.034; quality of life: *P* = 0.023; body image: *P* = 0.227; and self‐esteem: *P* = 0.032). However, as neither of the patients of the intention‐to‐treat sample received the intervention, results are presented with the per‐protocol sample to better evaluate the relative effectiveness of the intervention over WL control.
